# PD-L1 expression in tumor and inflammatory cells is associated with favorable tumor features and favorable prognosis in muscle-invasive urothelial carcinoma of the bladder not treated by immune checkpoint inhibitors

**DOI:** 10.1186/s12894-024-01482-z

**Published:** 2024-04-24

**Authors:** Henning Plage, Kira Furlano, Sebastian Hofbauer, Sarah Weinberger, Bernhard Ralla, Antonia Franz, Annika Fendler, Michela de Martino, Florian Roßner, Sefer Elezkurtaj, Martina Kluth, Maximilian Lennartz, Niclas C. Blessin, Andreas H. Marx, Henrik Samtleben, Margit Fisch, Michael Rink, Marcin Slojewski, Krystian Kaczmarek, Thorsten Ecke, Steffen Hallmann, Stefan Koch, Nico Adamini, Henrik Zecha, Sarah Minner, Ronald Simon, Guido Sauter, Joachim Weischenfeldt, Tobias Klatte, Thorsten Schlomm, David Horst, Simon Schallenberg

**Affiliations:** 1 Department of Urology, Charité – Universitätsmedizin Berlin, Corporate Member of Freie Universität Berlin, Humboldt-Universität zu Berlin and Berlin Institute of Health, Berlin, Germany; 2 Institute of Pathology, Charité – Universitätsmedizin Berlin, Corporate Member of Freie Universität Berlin, Humboldt-Universität zu Berlin and Berlin Institute of Health, Berlin, Germany; 3 Institute of Pathology, University Medical Center Hamburg-Eppendorf, Martinistr. 52, 20246 Hamburg, Germany; 4 Department of Pathology, Academic Hospital Fuerth, Fuerth, Germany; 5 Department of Urology, University Medical Center Hamburg-Eppendorf, Hamburg, Germany; 6 Department of Urology, Marienhospital Hamburg, Hamburg, Germany; 7 Department of Urology and Urological Oncology, Pomeranian Medical University, Szczecin, Poland; 8 Department of Urology, Helios Hospital Bad Saarow, Bad Saarow, Germany; 9 Department of Pathology, Helios Hospital Bad Saarow, Bad Saarow, Germany; 10 Department of Urology, Albertinen Hospital, Hamburg, Germany; 11 Biotech Research & Innovation Center (BRIC), University of Copenhagen, Copenhagen, Denmark; 12 Finsen Laboratory, Rigshospitalet, Copenhagen, Denmark

**Keywords:** PD-L1, Urothelial bladder carcinomas, Tissue microarray, Immunohistochemistry

## Abstract

**Background:**

A high level of PD-L1 expression is the most relevant predictive parameter for response to immune checkpoint inhibitor (CPI) therapy in urinary bladder cancer. Existing data on the relationship between PD-L1 expression and the natural course of disease are controversial and sparse.

**Methods:**

To expand our understanding of the relationship between PD-L1 expression and parameters of cancer aggressiveness, PD-L1 was analyzed on tissue microarrays containing 2710 urothelial bladder carcinomas including 512 patients with follow-up data who underwent radical cystectomy and follow-up therapies in the pre-immune checkpoint inhibitor therapy era.

**Results:**

Tumor cell positivity in ≥10% of cells were seen in 513 (20%) and an immune cell positivity occurred in 872 (34%) of 2566 interpretable cancers. PD-L1 positivity in tumor cells increased from pTaG2 low grade (0.9% positive) to pTaG3 high grade (4.1%; *p* = 0.0255) and was even higher in muscle-invasive (pT2–4) carcinomas (29.3%; *p* < 0.0001). However, within pT2–4 carcinomas, PD-L1 positivity was linked to low pT stage (*p* = 0.0028), pN0 (p < 0.0001), L0 status (*p* = 0.0005), and a better prognosis within 512 patients with cystectomy who never received CPIs (*p* = 0.0073 for tumor cells and *p* = 0.0086 for inflammatory cells). PD-L1 staining in inflammatory cells was significantly linked to PD-L1 staining in tumor cells (*p* < 0.0001) and both were linked to a positive p53 immunostaining (p < 0.0001).

**Conclusion:**

It cannot be fully excluded that the strong statistical link between PD-L1 status and favorable histological tumor features as well as better prognosis could influence the outcome of studies evaluating CPIs in muscle-invasive urothelial carcinoma.

**Supplementary Information:**

The online version contains supplementary material available at 10.1186/s12894-024-01482-z.

## Background

Urothelial carcinoma of the urinary bladder belongs to the ten most common malignant cancer types worldwide [1]. About 80% of patients present with non-invasive low-grade (pTa) or minimally invasive (pT1) stage urothelial bladder carcinomas. These carcinomas are characterized by a good prognosis and can be treated by transurethral resection. The treatment of patients with muscle-invasive urothelial carcinomas usually involves radiotherapy or radical cystectomy and neoadjuvant chemotherapy. Outcome of these patients is variable, but almost 50% develop metastasis and eventually die from their disease [2].

As in many other cancer types, immune checkpoint inhibitors (CPI) have become increasingly important for bladder cancer treatment (summarized in [3]). Atezolizumab, Nivolumab, Pembrolizumab, Avelumab, and Durvalumab have all been FDA approved for treatment of locally advanced or metastatic urothelial carcinoma of the bladder and the upper urinary tract (summarized in [4]). Although CPI are effective in metastatic urothelial bladder cancer, just a small proportion of treated patients will find a clear benefit while a high number of patients will be exposed to potentially significant side effects and toxicity with little improvement of quality of life or survival (summarized in [5]). A high level of programmed cell death 1 Ligand 1 (PD-L1) expression in cancers is the most relevant predictive parameter for response to CPI therapy and therefore used for decision making on whether or not CPI treatment is given [6]. In this context, the relationship of PD-L1 expression with tumor progression or disease outcome in patients not treated by CPIs is of interest. A significant link between PD-L1 expression and the natural history of the disease could influence the perception on the therapeutic success of CPI therapy. Data on this subject are controversial. At least 13 studies on 36–248 patients have analyzed the relationship between PD-L1 expression in either tumor cells or immune cells and parameters of urothelial cancer aggressiveness that were unrelated to CPI therapy and found links between high PD-L1 expression and favorable tumor features [7], unfavorable features [8–13] or failed to find relationships to prognostic parameters [14–19].

In an attempt to further expand our understanding of the relationship between PD-L1 expression and cancer aggressiveness, we investigated PD-L1 expression by immunohistochemistry (IHC) in a large cohort of 2710 bladder cancer patients including 512 patients with follow-up data who underwent radical cystectomy and follow-up therapies in the pre-CPI era. For optimal standardization, all analyses were done in a tissue microarray (TMA) format.

## Materials and methods

### Tissue microarrays (TMA)

The TMA method allows the analysis of a large number of molecular-genetic alterations on one TMA set. The TMAs used in this study were first employed in a study on the prognostic role of GATA3 expression in bladder cancer [20]. The TMA set were constructed from one 0.6 mm sample each from 2710 urothelial bladder tumors. The tumors were collected from the Institute of Pathology, University Hospital Hamburg, Germany, Institute of Pathology, Charité Berlin, Germany, Department of Pathology, Academic Hospital Fuerth, Germany, or Department of Pathology, Helios Hospital Bad Saarow, Germany, and/or treated at Department of Urology, University Hospital Hamburg, Germany, Department of Urology, Charité Berlin, Germany, Department of Urology, Helios Hospital Bad Saarow, Germany, Department of Urology, Albertinen Hospital, Hamburg, Germany, and Department of Urology and Urological Oncology, Pomeranian Medical University, Szczecin, Poland. Patients with pTa/pT1 disease were treated by transurethral bladder tumor resection with or without postoperative or adjuvant instillation therapy. Patients with pT2 carcinomas on the biopsy were treated by radical cystectomy, out of which 462 were pT2, 615 were pT3, and 298 were pT4. Available clinical follow up data and histopathological data were grade, tumor stage (pT), status of venous (V) and lymphatic (L) invasion, and lymph node status (pN) (Table 1), as well as clinical follow up data (overall survival; OS: time between cystectomy and death) from 512 patients with pT2–4 carcinomas treated by cystectomy (median: 13 months; range: 1–141 months) who had either died or appeared for their last follow-up visit before the approval of the first CPI for bladder cancer therapy (2017) in Germany and in Poland. The grading of pTa tumors included both a classification according to WHO 2004 [21] and Mostofi 1973 [22] which were valid at the time of the respective diagnoses. A centralized review of the cases was not done. Data on p53 immunostaining were available from a previous study [23]. The tissues were fixed in 4% buffered formalin and then embedded in paraffin. The TMA manufacturing process has previously been described in detail [24, 25]. The use of archived remnants of diagnostic tissues for TMA manufacturing, their analysis for research purposes, and patient data were according to local laws (HmbKHG, §12, article 1) and analysis had been approved by the local ethics committee (Ethics commission Hamburg, WF-049/09). All work has been carried out in compliance with the Helsinki Declaration.

**Table 1:** Patient cohort

	study cohort on TMA (***n*** = 2710)
**follow up**	512
months	
mean	19.3
median	13.0
**pathological tumor stage**	
pTa	887 (39.2%)
pT2	462 (20.4%)
pT3	615 (27.2%)
pT4	298 (13.2%)
**tumor grade**	
G2	820 (30.6%)
G3	1858 (69.4%)
**pathological lymph node status**	
pN0	734 (62.0%)
pN+	449 (38.0%)
**resection margin status**	
R0	595 (80.6%)
R1	143 (19.4%)
**lymphatic vessel infiltration**	
L0	275 (49.5%)
L1	281 (50.5%)
**blood vessel infiltration**	
V0	450 (74.4%)
V1	155 (25.6%)

Percent in the column “study cohort on TMA” refers to the fraction of samples across each category. Numbers do not always add up to 2710 in the different categories because of cases with missing data

### Immunohistochemistry

For this study we used identical methods for immunohistochemical evaluation of PD-L1 as previously described [26]. Freshly cut TMA sections were immunostained in one experiment and 1 day. Slides were deparaffinized with xylol, rehydrated through a graded alcohol series, and exposed to heat-induced antigen retrieval for 5 minutes in an autoclave at 121 °C in a pH 7.8 Tris-EDTA-Citrat (TEC) puffer, Endogenous peroxidase activity was blocked with Dako REAL Peroxidase-Blocking Solution (Agilent Technologies, Santa Clara, CA, USA; #S2023) for 10 minutes. Primary antibody specific against PD-L1 protein (recombinant rabbit monoclonal, MS Validated Antibodies, Hamburg, Germany, clone MSVA-711R; #2083-711R) was applied at 37 °C for 60 minutes at a dilution of 1:150. Bound antibody was then visualized using the EnVision Kit (Agilent Technologies, Santa Clara, CA, USA; #K5007) according to the manufacturer’s directions. The sections were counterstained with hemalaun. In tumor cells, a cut-off of ≥10% positive tumor cells defined PD-L1 positivity. In immune cells, PD-L1 staining was semiquantitatively assessed as “negative” (no staining), “few positive” (few cells stained), and “many positive” (many cells stained). Examples of these categorize are shown in supplementary Fig. 1.

### Statistics

Statistical calculations were performed with JMP16® software (SAS®, Cary, NC, USA). Contingency tables were created, and Chi^2^-tests were performed to test for associations between pathological or other molecular parameters and PD-L1 immunostaining. Overall survival curves were calculated according to Kaplan-Meier. The Log-Rank test was applied to detect significant differences between groups and *p*-value of ≤0.05 was assumed to be statistically significant.

## Results

### Technical issues

A total of 2566 (95%) of 2710 urothelial carcinomas were interpretable for PD-L1 immunostaining. Non-informative spots were caused by a lack of unequivocal tumor cells on the TMA spots or absence of entire tissue spots on the TMA.

### PD-L1 immunohistochemistry in urothelial carcinomas

**Fig. 1:**
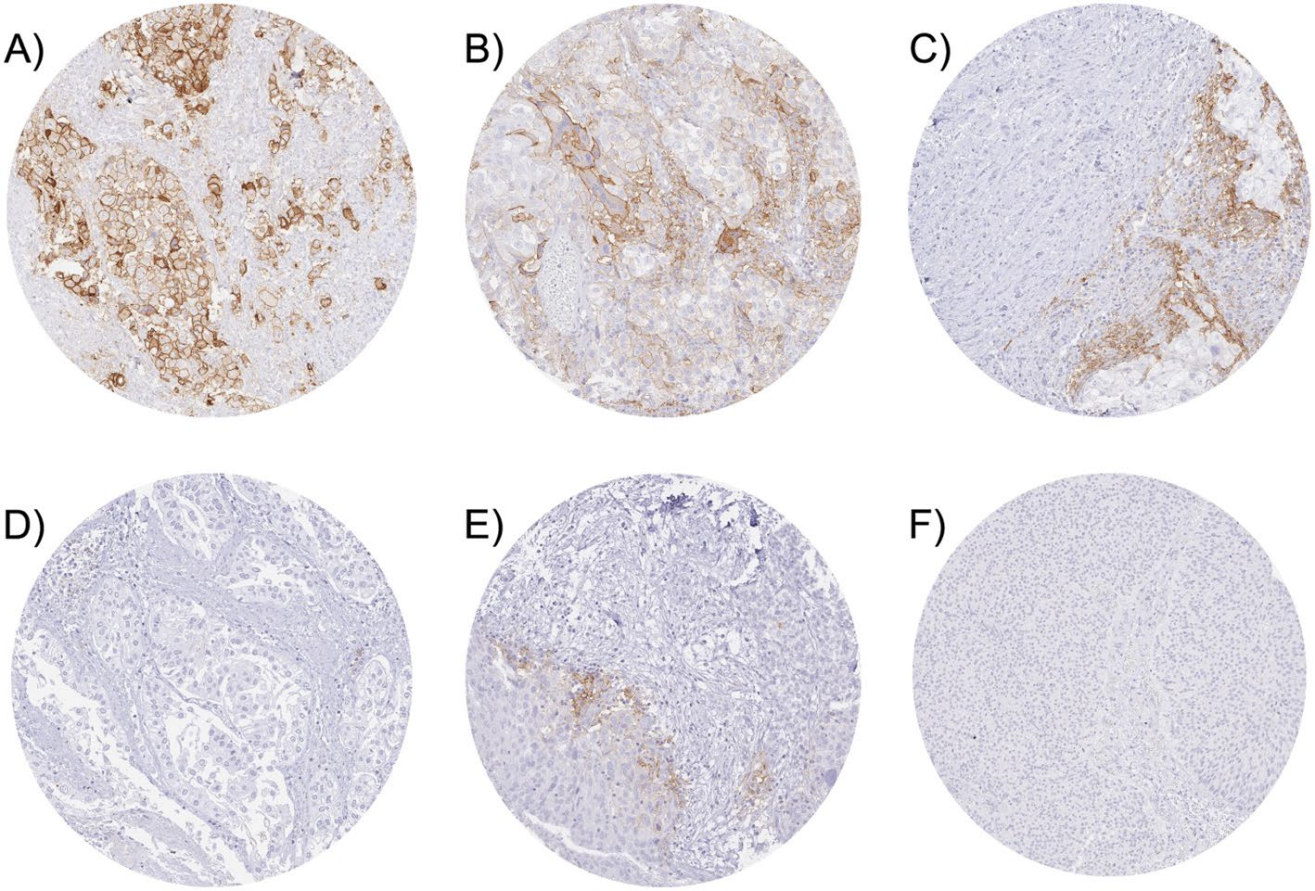
PD-L1 expression in urothelial carcinomas. The panels show pT2–4 urothelial carcinomas with strong PD-L1 staining in tumor cells only (**A**), weak PD-L1 staining in tumor cells and prominent positivity in immune cells (**B**), strong PD-L1 staining in immune cells only (**C**), and complete absence of PD-L1 staining (**D**). Examples of pTa tumors show a prominent PD-L1 positivity of peritumoral macrophages (**E**) and absence of PD-L1 staining (**F**)

A membranous staining in ≥10% of cancer cells was seen in 513 (20%) and immune cell staining was seen in 872 (34%) of the 2566 (822 pTa and 1744 pT2–4) interpretable cancers. Representative images are given in Fig. 1.

**Fig. 2:**
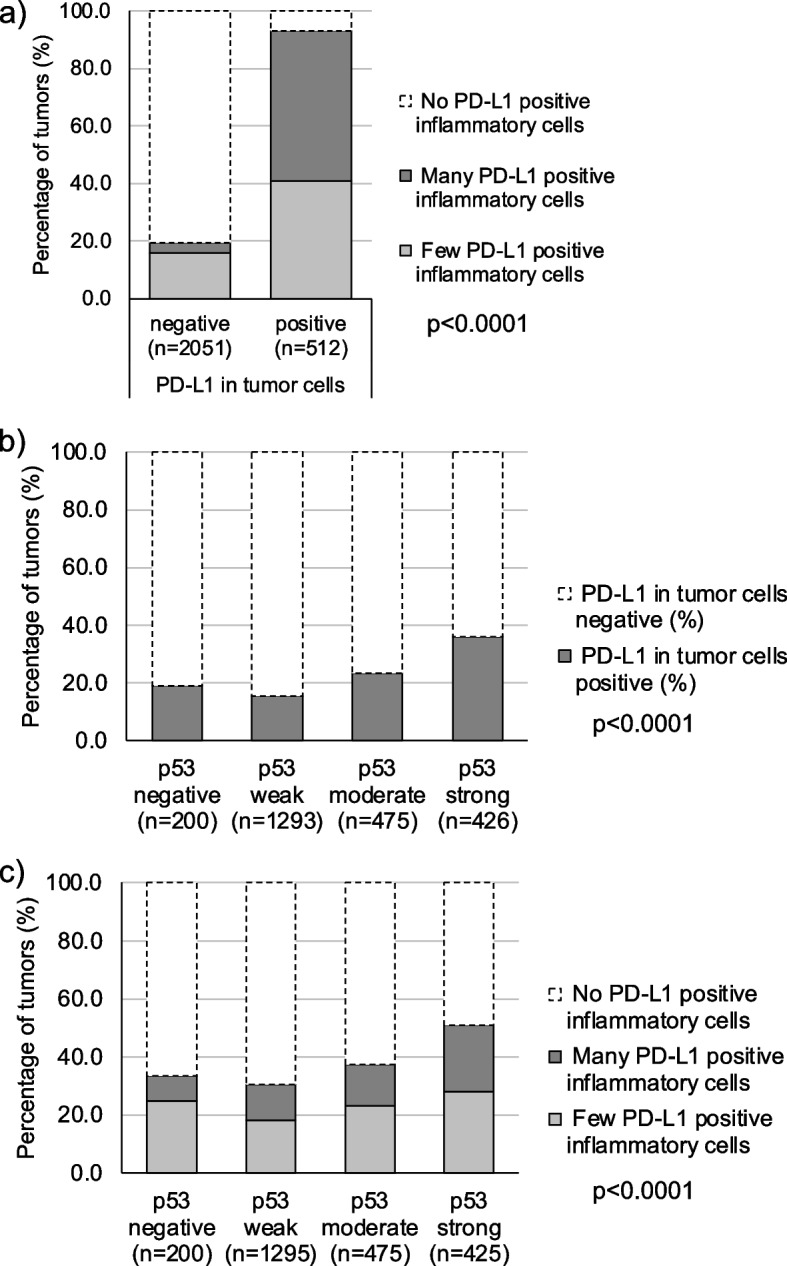
PD-L1 in tumor and inflammatory cells and p53 immunostaining. **a** PD-L1 in tumor vs inflammatory cells, **b** PD-L1 in tumor cells vs p53, and **c** PD-L1 in inflammatory cells vs p53

Associations between PD-L1 tumor and immune cell staining and histopathological tumor parameters are shown in Table 2. The rate of PD-L1 positivity in tumor cells increased from pTaG2 low grade (0.9% positive) to pTaG2 high grade (3.2%) and to pTaG3 (4.1%; *p* = 0.0255), and was even higher in muscle-invasive (pT2–4) carcinomas (2.1% positive for all pTa vs. 29.3% for pT2–4; *p* < 0.0001). PD-L1 positivity was associated with higher grade in pT2–4 carcinomas (*p* = 0.0154). However, in these muscle-invasive cancers both tumoral and inflammatory cell PD-L1 positivity was linked to low pT stage (*p* = 0.0028 for tumoral cells / *p* = 0.0013 for inflammatory cells), absence of nodal metastasis (*p* < 0.0001 each), and absence of lymphangiosis carcinomatosa (*p* = 0.0005 / *p* = 0.0419). PD-L1 staining in inflammatory cells was significantly linked to PD-L1 staining in tumor cells (Fig. 2a, *p* < 0.0001). Accordingly, PD-L1 positivity in inflammatory cells was higher in pT2–4 (44.8%) than in pTa tumors (12.5%; p < 0.0001; Table 2). PD-L1 positivity in tumor cells and in inflammatory cells was also significantly associated with a positive p53 immunostaining (p < 0.0001 each; Fig. 2b-c).

**Table 2:** PD-L1 immunostaining in tumor and inflammatory cells and cancer phenotype

		PD-L1 in tumor cells				PD-L1 in inflammatory cells			
	n	negative (%)	positive (%)	P	n	negative (%)	few positive (%)	many positive (%)	P
All cancers	2566	80	20		2566	65.9	20.7	13.4	
pTa G2 low	452	99.1	0.9	0.0255	452	89.2	5.5	5.3	0.2181
pTa G2 high	222	96.8	3.2		222	83	8	8.9	
pTa G3	148	95.9	4.1		148	89.2	6.1	4.7	
pT2	437	66.6	33.4	0.0028	437	51.6	29.4	19	0.0013
pT3	582	70.3	29.7		582	52.9	31.1	16	
pT4	280	78.2	21.8		280	65.4	23.9	10.7	
G2	100	81	19	0.0154*	100	66	22	12	0.0768*
G3	1172	70	30		1171	54.4	29.2	16.4	
pN0	634	65.6	34.4	<0.0001*	634	51.7	29.4	19	<0.0001*
pN+	425	79.3	20.7		425	62.1	28	9.9	
R0	524	67.2	32.8	0.0357*	524	53.1	29.8	17.2	0.0648*
R1	128	76.6	23.4		128	63.8	25.2	11	
L0	236	64.8	35.2	0.0005*	236	51.5	31.1	17.4	0.0419*
L1	255	78.8	21.2		255	62.7	23.9	13.3	
V0	399	68.7	31.3	0.1539*	399	53.5	29.4	17.1	0.2662*
V1	140	75	25		140	61.4	24.3	14.3	

* only in pT2–4 carcinomas

*pT* pathological stage, *G* Grade, *pN* pathological lymph node status, *R* resection margin status, *L* lymphatic vessel infiltration, *V* blood vessel infiltration

**Fig. 3:**
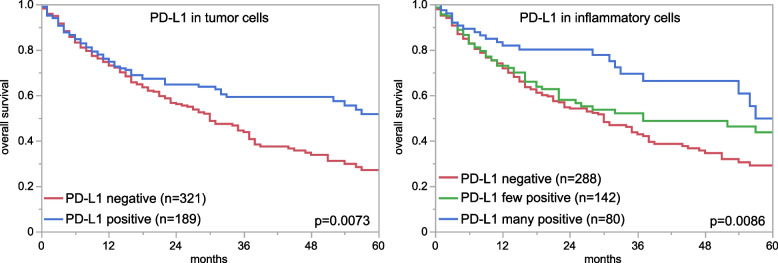
PD-L1 in tumor and inflammatory cells and prognosis in muscle-invasive urothelial carcinomas not treated by CPI

To estimate the impact of PD-L1 immunostaining in tumor and inflammatory cells on patient prognosis we analyzed the relationship between PD-L1 immunostaining and OS (time between cystectomy and death) in 512 patients with muscle-invasive carcinomas who had never been treated with CPIs. In these analyses, both PD-L1 positivity in inflammatory and tumor cells was significantly linked to a prolonged OS in comparison to patients with PD-L1 negative tumor and inflammatory cells (*p* = 0.0073 for tumor cells and *p* = 0.0086 for inflammatory cells; Fig. 3).

## Discussion

Our analysis of PD-L1 staining in more than 2500 urothelial carcinomas provided seemingly controversial results. PD-L1 positivity in both tumor cells and inflammatory cells was linked to high grade of malignancy and invasive tumor growth but – within muscle-invasive cancer – strongly related to features of less aggressive tumor behavior and a better patient prognosis estimated by a longer overall survival.

Earlier studies investigating PD-L1 expression in tumor cells have often focused on muscle-invasive (pT2–4) urothelial carcinomas. Our positivity rate of 29% in pT2-pT4 tumors is in the lower range of published data where PD-L1 positivity ranged from 9 to 88% in studies analyzing 10 to 936 pT2–4 carcinomas [26–29]. Reasons that are typically considered for discrepant results in IHC studies include different antibodies, staining protocols, and criteria to define positivity [30]. However, for PD-L1, multiple studies have shown that the most commonly used antibodies can all result in similar data within studies [31–34] and that even the use of laboratory developed PD-L1 tests yield similar results as Food and Drug Administration (FDA) approved companion diagnostics [30]. For the PD-L1 assay used in this study, we had earlier demonstrated a similar staining behavior as for several established PD-L1 antibodies such as clones E1L3N, SP142, and SP263 [26]. It is of note that a high variability of PD-L1 data also exists in studies using the same antibodies [15, 29]. The quantity of tissue analyzed per patient, section age and difficulties in the distinction of intraepithelial or peri-epithelial PD-L1 positive macrophages from true PD-L1 positive cancer cells might also contribute to the data diversity of PD-L1 IHC in the literature [26, 35, 36].

Our rate of 45% of muscle-invasive urothelial carcinomas with detectable PD-L1 positivity in tumor associated immune cells is also in the lower range of the 33 to 74% reported in the literature [7, 26]. Whether PD-L1 expression is more relevant if it occurs in tumor cells or in immune cells is unresolved [37–39]. The significant association between PD-L1 positivity in tumor cells and high levels of PD-L1 positive macrophages demonstrates that both mechanisms for immune cell suppression are commonly activated together. Their similar associations with histopathological features also suggest that the biological effect of PD-L1 is not highly dependent of the PD-L1 positive cell types.

PD-L1 positivity in tumor cells was significantly linked to favorable tumor phenotype and better prognosis in pT2–4 carcinomas. This observation is controversial to most findings from earlier studies. Associations of PD-L1 staining in tumor cells with pT, pN, L-status or clinical outcome have been analyzed in at least 8 cohorts of muscle-invasive urothelial cell carcinomas [7, 8, 10, 14, 40–43]. Only one of these studies have identified significant associations between PD-L1 positivity and favorable tumor parameters (low pT and absence of nodal metastasis) in a cohort of 139 urothelial carcinomas [7]. One additional study showed prolonged overall survival for tumors with PD-L1 positive tumor cells in a subset of 156 pTa to pT4 carcinomas that were not treated by CPI [44]. However, five other studies did not find a relationship between PD-L1 status and tumor aggressiveness in cohorts of 64 to 96 carcinomas [14, 40–43], and two further studies even reported a link between high PD-L1 levels and histological parameters of aggressive urothelial cancers in 236 and 248 carcinomas [8, 10]. It is of note that PD-L1 positivity has also been linked to favorable tumor features and good prognosis (in the absence of CPI therapy) in other tumor entities such as for example oral and lung squamous cell carcinomas [45, 46], and gastric cancer [47]. In contrast, most studies on kidney [48], breast [49], lung [50], and colorectal cancer [51] have linked PD-L1 positivity to adverse prognosis and unfavorable tumor features.

The strong association between p53 positivity and PD-L1 staining found in this study is in line with reports describing a similar link in other tumor entities such as for example triple negative breast cancer [52], endometrial cancer [53], hepatocellular carcinoma [54], and oral squamous cell carcinomas [55]. p53 is known to play an important role in DNA damage pathways, which also belong to the relevant mechanisms inducing up-regulation of PD-L1 expression (summarized in [56]). Cortez et al. [57] suggested a possible mechanism, as they described an influence of p53 on PD-L1 expression via the microRNA miR-34 and observed that tumors with *TP53* mutations had low miR-34 expression but higher PD-L1 levels. Another potential explanation lies in the high level of genomic instability in *TP53* mutated tumors which is typically linked to high level nuclear atypia, a high mutation rate and – consequently – higher immunogenicity of tumor cells [58]. Elevated immunogenicity of tumor cells could lead to a relevant immune response that causes a lower pT stage and fewer metastases although a fraction of these tumors upregulate PD-L1 in an attempt to evade the immune response (summarized in [59]). Such a scenario could also explain the seemingly paradoxical link of PD-L1 expression with high grade (severe nuclear atypia are reflective of high genomic instability and high mutation rate) and low pT/pN stage, possibly caused by effects of the immune response which is only incompletely suppressed by tumoral PD-L1 expression.

The very low rate of PD-L1 positivity in tumor cells seen in pTa tumors (2% PD-L1 positive) in this study is consistent with results from our earlier study investigating PD-L1 expression across 118 different tumor entities, and data from Inman et al. [10, 26]. In these studies, on 44 and 426 pTa tumors PD-L1 staining was found in 2 and 7% of all pTa tumors. The much lower rate of PD-L1 positivity in non-invasive than invasive urothelial carcinomas may be due to the rather limited interaction of pTa tumor cells with the immune system, as these cells are separated from the tumor associated stroma by a basal membrane.

Our study also suffers from limitations. The number of tumors examined is still too small. Especially when investigating subgroups such as muscle-invasive tumors without CPI, a higher number of cases than 512 would be desirable. Further limitations are the lack of information’s about additional tumor treatment (e.g. adjuvant or neoadjuvant chemotherapy), the retrospective nature of our analysis, and the absence of standardized continuous clinical follow-up evaluation of our patients. Future international collaborative studies should be designed to evaluate the role of PD-L1 in larger cohorts of urothelial carcinomas.

## Conclusion

The data of this large-scale study demonstrate a seemingly paradoxical link between PD-L1 positivity, high grade, and invasive tumor growth while PD-L1 positivity is tightly related to favorable tumor features and better prognosis within the clinically most relevant group of pT2–4 carcinomas. Therefore, it cannot be fully excluded that a link between PD-L1 status and the natural course of the disease may potentially influence the outcome of studies evaluating CPIs.

### Supplementary Information

**Supplementary Material 1.**

## Data Availability

The datasets generated and/or analyzed during the current study are available from the corresponding author on request.

## Abbreviations

CPI: Immune checkpoint inhibitor
FDA: Food and Drug Administration
IHC: Immunohistochemistry
L: Lymphatic invasion
pT: Pathological tumor stage
TMA: Tissue microarray
OS: Overall survival
PD-L1: Programmed cell death 1 Ligand 1
pN: Pathological lymph node status
V: Venous invasion
