# Supplementary material for: PD-L1 expression in tumor and inflammatory cells is associated with favorable tumor features and favorable prognosis in muscle-invasive urothelial carcinoma of the bladder not treated by immune checkpoint inhibitors
Source: BMC Urol. 2024 Apr 24;24:96. doi: 10.1186/s12894-024-01482-z (PMC11041044; doi:10.1186/s12894-024-01482-z)
Supplement: Supplementary file 1 — Supplementary Material 1. [file 12894_2024_1482_MOESM1_ESM.pptx]

## Slide 1
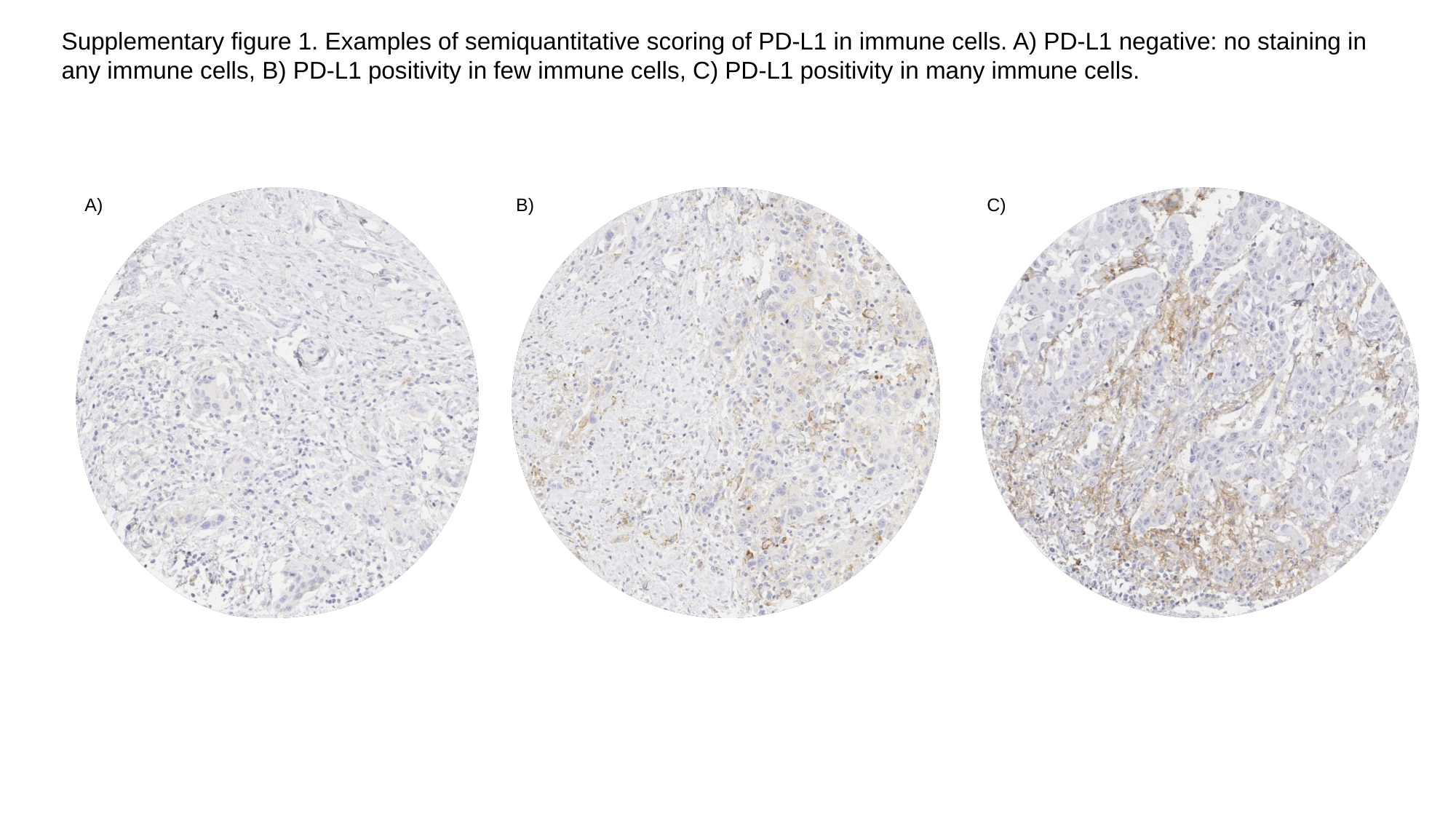

Supplementary figure 1. Examples of semiquantitative scoring of PD-L1 in immune cells. A) PD-L1 negative: no staining in any immune cells, B) PD-L1 positivity in few immune cells, C) PD-L1 positivity in many immune cells.
A)
B)
C)
